# Oral Lesions Associated with COVID-19 and the Participation of the Buccal Cavity as a Key Player for Establishment of Immunity against SARS-CoV-2

**DOI:** 10.3390/ijerph191811383

**Published:** 2022-09-09

**Authors:** Jose Roberto Gutierrez-Camacho, Lorena Avila-Carrasco, Maria Calixta Martinez-Vazquez, Idalia Garza-Veloz, Sidere Monserrath Zorrilla-Alfaro, Veronica Gutierrez-Camacho, Margarita L. Martinez-Fierro

**Affiliations:** Molecular Medicine Laboratory, Unidad Académica de Medicina Humana y Ciencias de la Salud, Universidad Autónoma de Zacatecas, Zacatecas 98160, Mexico

**Keywords:** COVID-19, cavity buccal, oral lesion

## Abstract

Background: Some oral lesions have been described in patients infected with severe acute respiratory syndrome coronavirus 2 (SARS-CoV-2); the possibility has been raised that the buccal lesions observed in patients with the coronavirus disease 2019 (COVID-19) are due to this virus and the patient’s systemic condition. The aim of this review was to integrate the knowledge related to the oral lesions associated with COVID-19 and the participation of the buccal cavity in the establishment of immunity against SARS-CoV-2. Methods: A literature search on the manifestations of buccal lesions from the beginning of the pandemic until October 2021 was carried out by using the PubMed database. A total of 157 scientific articles were selected from the library, which included case reports and reports of lesions appearing in patients with COVID-19. Results: Oral lesions included erosions, ulcers, vesicles, pustules, plaques, depapillated tongue, and pigmentations, among others. The oral cavity is a conducive environment for the interaction of SARS-CoV-2 with the mucosal immune system and target cells; direct effects of the virus in this cavity worsen the antiviral inflammatory response of underlying oral disorders, immunodeficiencies, and autoimmunity primarily. Conclusions: The oral cavity is an accessible and privileged environment for the interaction of SARS-CoV-2 with the mucosal immune system and target cells; the direct effects of the virus in this cavity worsen the antiviral inflammatory response of underlying oral disorders, in particular those related to immunodeficiencies and autoimmunity.

## 1. Introduction

The coronavirus disease 2019 (COVID-19) is the pathology caused by severe acute respiratory syndrome coronavirus 2 (SARS-CoV-2), which is a virus containing in its genetic material a single strand of RNA [1]. Some of the most common clinical signs and symptoms of COVID-19 are fever, sore throat, headache, shortness of breath, dry cough, belly pain, vomiting, and sometimes diarrhea [2]. Angiotensin-converting enzyme receptor 2 (ACE2) is one of the main known receptors for SARS-CoV-2 to enter the cells of the lungs, liver, kidney, gastrointestinal system, and even on the endothelia of dermal papillary vessels and on the epithelial surfaces of sweat glands [3]. Various skin manifestations have been described in patients with COVID-19, including pseudo-chilblain, varicelliform lesions, erythema multiforme-like lesions, urticaria form, maculopapular, purpura and petechiae, mottling, and livedo reticularis-like lesions [4,5]. In the oral cavity, ACE2 is expressed in the oral mucosa, especially and in greater quantity in the lingual surface and saliva-producing glands in relation to the mucosa of the mouth or palate [6]. Dysgeusia is the first recognized oral symptom of COVID-19 reported in 38% of patients, especially in North Americans and Europeans and patients with mild–moderate disease severity [4]. Since the first oral manifestations associated with COVID-19 were described, several reports have been published describing a wide variety of lesions, where the most frequent oral lesion manifestation is ulceration [7], in addition to white plaques, petechiae, geographic tongue, macules, nodules, bullous angina, necrotizing periodontal disease, blisters, and erythema-multiforme-like lesions [8,9]. It has been reported that loss of taste and/or smell remains for up to 14 days and progresses more rapidly in older patients; recovery from mouth lesions occurs at the same time as patients recover from COVID-19, representing an association between the manifestations of all clinical lesions appearing in the mouth and SARS-CoV-2 infection of patients [10].

Given that the clinical manifestations of COVID-19 beyond lung damage caused by inflammation are not yet adequately understood, the oral health conditions associated with COVID-19 continue to be studied to gain a better appreciation of the oral manifestations. The crisis that the world continues to face from COVID-19 has highlighted the importance of understanding the implicit conditions that lead to COVID-19-related outcomes, primarily mortality, as well as positivity and severity [11,12,13,14].

## 2. The Oral Cavity and Its Role in Immunity

The oral cavity has three major host defenses against microbial invasion: the oral mucosa, nonspecific (innate) immunity, and adaptive (acquired) immunity. The oral mucosa consists of a layer of interconnected epithelial cells containing mainly keratinocytes resting on a basal membrane and provides a physical barrier that protects the underlying tissues from microorganisms and environmental threats in the oral cavity [15,16,17]. Cells of the immune system and oral keratinocytes in the lamina propria of the oral mucosa detect some pathogen-associated molecular patterns that have been conserved by evolution in specific classes of microorganisms [18,19]. Pattern recognition receptors distinguish between different molecular structures of microorganisms and thus prevent the generation of immunoinflammatory responses against these microorganisms [20,21]. Pattern recognition receptor families have been described in some reports, in which have been included for example the toll-like receptor family (TLR-1 to TLR-10) and the C-type lectin receptor family (Dectin-1, Dectin-2, dendritic cell specific intercellular adhesion molecule 3-grabbing nonintegrin) [22]. The initiation and determination of the type of specific adaptive immune responses induced by pattern recognition receptors also dictate the magnitude and duration of the responses and whether or not memory T cells are activated [20]. The specificity, type, and sensitivity of pattern recognition receptor-mediated adaptive immune responses are determined by the nature of the infectious agent, for example, if they are viruses, bacteria, fungi, and/or protozoan [23]. By the microenvironment characteristics, the type of cells expressing each family of pattern recognition receptors, the anatomical site where these cells are located, and the combination of interactions that occur between these factors [24]. Warning signals generated by some tissue-damaging factors, including hypoxia, radiation, and trauma to some extent affect the speed and magnitude of the immune response [25].

## 3. Oral Cavity during Viral and Bacterial Infections

Several viral diseases usually affect the oral cavity; for example, human immunodeficiency virus (HIV) infection may initially present with oral lesions, human papillomavirus (HPV) infection often increases the risk of developing oral squamous cell carcinoma, and oral damage has been documented during hepatitis B and C virus infections [26,27,28,29].

**Figure 1:**
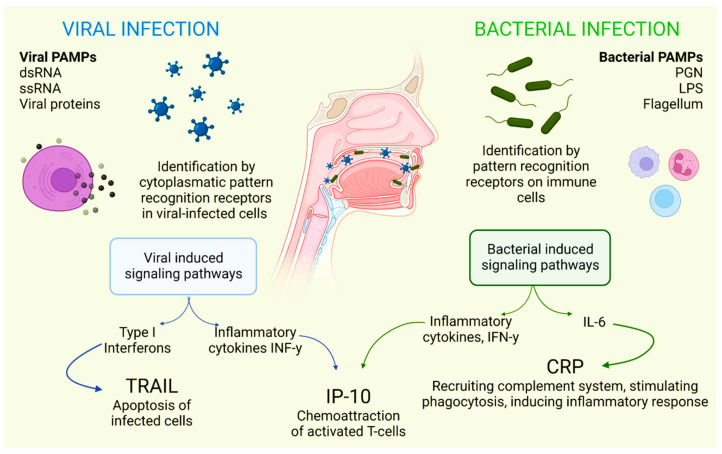
Selective host response against bacterial and viral infections. Different molecules and signaling pathways are dynamically involved in complementing the response to virus and bacterial infections (including CRP, IP-10 and TRAIL). IL-6: interleukin-6; LPS: lipopolysaccharide; PAMPs: pathogen-associated molecular patterns; PGN: peptidoglycan; ssRNA: single-stranded RNA; dsRNA: double-stranded RNA. Adapted with permission from Oved, K. et al. (2015) [32], which was distributed under the terms of the Creative Commons Attribution License https://creativecommons.org/licenses/by/4.0/; accessed date 28 November 2021.

There is a combination of proteins with complementary dynamics in virus and/or bacterial infection in the oral cavity. Tumor necrosis factor-related apoptosis-inducing ligand (TRAIL) has been shown to be significantly stimulated in response to infections that are primarily viral in origin [30]. Interferon gamma-induced protein-10 (IP-10) levels are generally slightly elevated in patients with bacterial infections and highly elevated in patients with viral infections. It is also known that C-reactive protein (CRP) levels are commonly found to be increased in patients who have developed infections of bacterial origin and that CRP levels are less elevated in patients who have developed infections caused by viruses (Figure 1) [31].

Some drugs used in the treatment of viral infections can also contribute to damage to the oral cavity. High doses of corticosteroids may trigger fungal infections such as oral candidiasis; antiviral drugs can cause dry mouth, aphthous ulcers, and stomatitis; and the use of antiviral drugs can cause dry mouth [33]. Additionally, many patients have been prescribed antibiotics that are effective against Gram-negative and Gram-positive bacteria, which usually has a direct impact on the homeostasis of the mouth and all microorganisms found in this cavity [34].

### Oral Cavity and SARS-CoV-2 Infection

As mentioned before, SARS-CoV-2 is an RNA-positive virus with an icosahedral morphology that possesses S proteins that are the binding site for ACE2 in humans [1], which, in addition to the lungs, pancreas, adipose tissue, liver, or kidney, this receptor is also expressed in salivary glands [35]. The oral cavity is a gateway for many pathogens, and SARS-CoV-2 is not the exception. This virus is detected in the saliva of all COVID-19 patients even with more sensitivity than that of nasopharyngeal testing [36].

**Figure 2:**
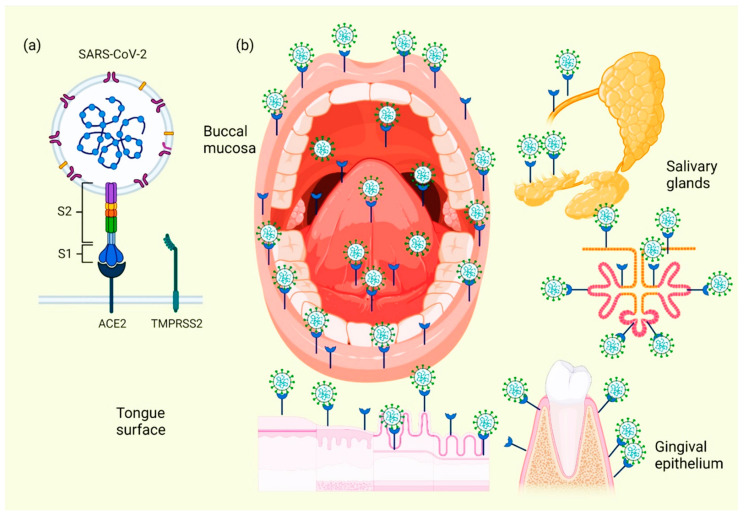
Location of ACE2 on oral cavity and its interaction with the SARS-CoV-2. Binding between the SARS-CoV-2 S protein and the ACE2 protein allows entry of the coronavirus, which subsequently allows its replication and the immediate activation of a possible innate immune response against the virus, including the infiltration of a myriad of immune cells and the subsequent production of many proinflammatory cytokines. This triggers the manifestation of symptoms and signs in the oral cavity of patients with COVID-19 (**a**). Various spaces and surfaces in the oral cavity where the virus and its receptors are detected, such as the oral mucosa, periodontal tissues, salivary glands, and tongue (**b**).

When the ACE2 protein of the host cell and the S protein of SARS-CoV-2 bind, an interaction occurs that allows the coronavirus to use the machinery of these host cells to replicate and subsequently destroy these same cells, triggering the oral symptoms and signs [37]. In addition to this mechanism, which explains the cause of several manifestations of oral lesions caused by COVID-19, it is also possible that these lesions are the result of opportunistic infections that facilitate immune system alterations and may also be facilitated by possible systemic damage and adverse effects that can be triggered by treatment [38]. Figure 2 represents the location of ACE2 in different tissues and structures of the oral cavity, as well as those interacting with SARS-CoV-2.

Cellular analyses of ACE2 expression as a SARS-CoV-2 entry factor revealed that no oral epithelial subpopulations are at particular risk. The ACE2 receptor was detected in nine oral epithelial cell groups, including basal 1–3, basal cyclic, salivary gland ducts, serous salivary glands, and mucous salivary glands, indicating that multiple oral epithelial cell subpopulations are prone to infection [39]. Coexpression of the most important entry factors ACE2 and TMPRSS2 in mucosal and salivary gland epithelial cells was rare in salivary gland acini and ducts [40]. The clinical development of chemosensitivity disorders usually occurs at the beginning of the infection phase in which the first symptoms are already present, usually within the first three days [41]. Two theories have been described on the pathophysiological factors causing dysgeusia and loss of olfaction in the course of COVID-19 infection: the first theory indicates that SARS-CoV-2 infects neurons using active cellular transport to gain access to the central nervous system [42]. The second theory suggests that these dysfunctions are due to the inhibition of the ACE2 receptor; inhibitors of this protein have already been reported to induce ageusia through a complex mechanism involving the sodium channel present in the taste buds and the G-protein-coupled receptor; upon interaction between SARS-CoV-2 and the host cell receptors, the latter are inactivated, resulting in the loss of chemical taste signaling in their action potentials and, therefore, of the correct sensory perception of taste [41].

## 4. Oral Manifestations in Patients with COVID-19

Alterations in smell and taste are some early indicators of SARS-CoV-2 infection and are effective in decision making, and dry mouth is an oral presenting symptom [43,44,45], infection of salivary ducts by SARS-CoV-2 has been experimentally confirmed in animal models, which explains the presence of acute sialadenitis, discomfort, pain, inflammation or even improper functioning of the saliva-producing glands [46].

Patients with COVID-19 experience fatigue, anosmia, fever, dysgeusia, and dry cough, in which later appeared a generalized erythema on the hard palate and oropharynx with pustules and petechiae on the edge of the soft palate; the suggested diagnosis was enanthem due to COVID-19 [47]. Petechiae on the palate, lower lip, and oropharyngeal mucosa due to COVID-19 infection were also observed [48]. Pigmentation in the attached and interpapillary gingiva due to increased levels of inflammatory cytokines and arachidonic acid metabolites secondary to the production of basic-fibroblast growth factor (bFGF) and stem cell factor (SCF) from basal layer keratinocytes has been reported in some SARS-CoV-2 patients [49]. In a case series reported by Carreras-Presas et al., patients suspected of being infected with SARS-CoV-2 presented fever, halitosis, submandibular lymphadenopathy, and oral lesions [3]. The oral lesions were common to that reported by Patel et al. and included erythematous, painful, diffuse, and edematous gingiva with necrosis of the interpapillary areas [3,50]. In spite of authors suggesting that the oral lesions observed were due to SARS-CoV-2 provokes exanthematic lesions that may resemble other viral processes [3], it has also been suggested that the lesions may be associated with bacterial coinfections in conjunction with COVID-19 [50]. Many patients with COVID-19 suffer from weakness, present with fever, have episodes of diarrhea, and occasionally have abdominal pain coinciding with the presence of an oral erythematous papular rash; lesions occurring in the oral cavity consist of erosions on the dorsum of the tongue and the mucosa of the mouth, as well as vesicular eruptions [51]. Table 1 lists some of the descriptive characteristics of representative studies evaluating oral manifestations in patients with COVID-19, including type of lesions, the number of patients (sample), study design, the country, and year in which each study was carried out.

### 4.1. Herpetiform Lesions

Herpetiform lesions present as multiple painful, unilateral round, yellowish-gray, unilateral ulcers with an erythematous border on nonkeratinized and keratinized mucosa. The expression of these lesions precedes the systemic symptoms. In one case, geographic tongue appeared after recovery from herpetiform lesions [9,51,62]. Clinically, it was observed that the presence of oral ulcerations was very varied, ranging from lesions similar to aphthous stomatitis to the presence of generalized ulcerations with necrosis, some of these oral lesions had a herpetiform pattern, with resemblance to herpes infection, but rendered a negative result to the test for herpes simplex virus (HSV) [58,63]. Immunosuppression and stress associated with COVID-19 were suggested as the cause for the appearance of secondary herpetic gingivostomatitis [64,65]. Patients suffering from acute COVID-19 infection, together with inadequate therapeutic measures, could contribute to adverse oral health outcomes [62].

### 4.2. Erosive or Ulcerative Lesions

Erosive or ulcerative lesions appeared as painful lesions with irregular borders on the tongue, labial mucosa, and hard palate [66,67]. The lesions appeared after a latency time of 4–7 days before the onset of systemic symptoms. Different factors were suggested as causes for the development of ulcerative and erosive lesions, such as drug eruption, thrombotic vasculopathy secondary to COVID-19, and vasculitis [53,68].

### 4.3. Red and White Plaques

Red and white plaques have also been reported on the gingiva, dorsum of the tongue, and palate of patients with COVID-19 [55]. Candidiasis due to long-term antibiotic therapy, deterioration of general condition, and decreased oral hygiene may be the cause of the red or white plaques [49].

### 4.4. Erythema-Multiforme-like Lesions

Erythema-multiforme-like lesions appeared as blisters, scaly gingivitis, erosions, and painful cheilitis with hemorrhagic crusting in patients with skin lesions on all four extremities [56,69].

### 4.5. Angina Bullosa-like Lesions

Some patients confirmed with COVID-19 also had angina bullosa-like lesions as asymptomatic erythematous-purpuric blisters without spontaneous bleeding on the hard palate and tongue [57,59].

Is important to note that patients with COVID-19 who are intubated undergo tracheostomy or assisted external ventilation [70]. These maneuvers cause hyposalivation, which could aggravate the already existing lesions of the oral cavity and could trigger bacterial aspiration pneumonia [71]. The main effect of COVID-19 on the oral mucosa is multifactorial, the prominent role of the immune system is unavoidable, and the certain state of deregulation of this system may lead to infections or the risk of more severe autoimmune conditions through cytokine storm [72]. Some researchers claim that oral lesions contributing to COVID-19 are triggered by an inflammatory response that induces vascular inflammation [73]. Inflammation plays an important role in disease progression and clinical outcomes. Inappropriately increased inflammation causes a cytokine storm, leading to tissue damage, diffuse alveolar damage, myocarditis, renal failure, central nervous system involvement, and multiorgan failure, and is sometimes associated with a poor clinical prognosis. Coronavirus infection causes a release of proinflammatory cytokines, associated with a systemic inflammatory response syndrome (SIRS) [74], resulting in markedly elevated levels of interleukins (IL), which are usually: IL-2, IL-6, IL-7, granulocyte colony-stimulating factor, monocyte chemoattractant protein 1 (MCP-1), macrophage inflammatory protein 1 alpha (MIP1-a), interferon-inducible protein 10-γ (IP-10), and tumor necrosis factor (TNF)-alpha [75,76,77].

## 5. Periodontal Disease: A Risk Factor Associated with the Development of Chronic Diseases and Negative Outcomes for Human Health

Periodontal disease is one of the most common inflammatory pathologies representing the sixth most prevalent (20–50%) and represents a leading cause of tooth loss and masticatory dysfunction in adults. It is an infectious disease characterized mainly by progressive degradation of the periodontium due to inflammation [50,78]. The severity of periodontal disease increases significantly and gradually with age, showing a steady increase between the ages of thirty and forty [79]; this is a consequence of inefficient oral hygiene, which causes bacteria in dental plaque to trigger a local inflammatory reaction, and also the recruitment of neutrophils and many other inflammation-promoting cells that are mediated by proinflammatory cytokines [80,81]. Therefore, the main cause of periodontal destruction is the host immune defense against pathogens; in addition, periodontal bacteria also have the potential to access the bloodstream and spread systemically [82,83]. When some distant organs are already colonized, oral bacteria can contribute to triggering disease in these organs [84,85]. In established periodontitis, the gingival epithelial barrier is damaged, resulting in ulceration. Thus, the anatomical proximity between the sublingual Gram-negative bacteria and the blood stream facilitates their systemic spread; transient bacteremia may also occur after tooth brushing, chewing, flossing, and some dental office procedures [86,87,88].

**Figure 3:**
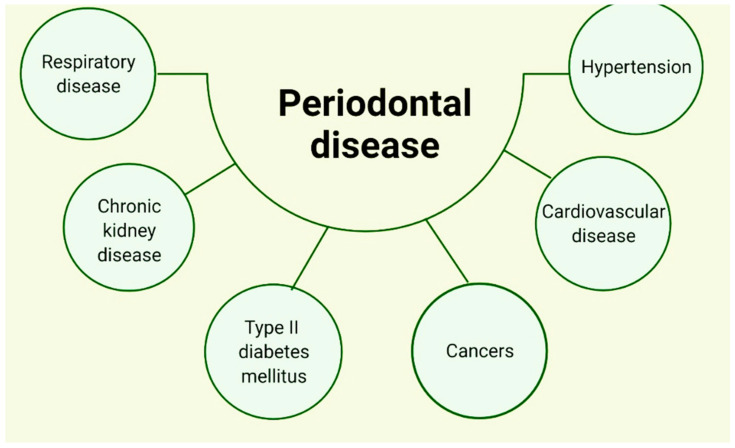
Relationship between different systemic pathologies and periodontal disease.

Importantly, *Aggregatibacter actinomycetemcomitans* (*A.* *actinomycetemcomitans*) and *Porphyromonas gingivalis* (*P. gingivalis*) are key microorganisms implicated in the pathogenesis of periodontal disease and have been identified in atherosclerotic plaques in humans [89]. The presence of *P. gingivalis* has also been identified in the brain tissue of patients with Alzheimer’s disease [85]. Different mechanisms have already been suggested to describe the translocation of oral bacteria to the respiratory system; saliva aspiration seems to be the most prominent mechanism. A group of oral microorganisms live in saliva, such as some bacteria, which are shed from some of the surfaces of the oral cavity; it can reflect changes in sub- and supragingival bacteria and thus function as a reservoir for multiple pathogens [90]. Several reports have already described that periodontitis affects overall systemic health [91,92], and, therefore, has been associated with an increased risk of many chronic diseases [93], mainly pathologies related to the heart [94,95,96], diabetes mellitus [97,98], high blood pressure [99], chronic disease of one or both kidneys [100], chronic obstructive pulmonary disease (COPD) and pneumonia [101], and cancer (Figure 3) [102]. These associations have been explained by the presence of environmental and genetic risk factors, as well as by the presence of chronic inflammation [103]. It has already been reported that periodontal disease can lead to an increased risk of cardiovascular disease of 19%, and there may even be an increased relative risk of up to 44% if patients with periodontitis are over 65 years of age [104]. Comparative studies have been conducted in patients who have shown a significantly higher prevalence of periodontal disease in patients with COPD compared to healthy individuals [101,105]. DM is clearly a systemic risk factor for periodontal disease, and it generally plays a determining role in the onset and progression of the disease itself, as it is associated with periodontal ligament degeneration, which leads to tooth loss [106,107].

A relationship has been suggested between chronic kidney disease (CKD) and periodontal disease; it has been concluded that periodontitis is a risk factor for CKD and that periodontal treatment produces positive outcomes in people with CKD [108,109]. Research on the subject has determined an increased risk of cancer due to periodontitis. It has been described that the risk of lingual cancer in some reports has increased up to 5.23 times and there is also a loss of several millimeters of alveolar bone. A correlation has also been reported although of lesser risk of periodontitis and esophageal, oral, pancreatic, and gastric cancers [110,111,112]. There are clinical and experimental reports that have suggested the relevance of inflammation at the time when an increase in blood pressure begins to occur; mainly, it has been elucidated that alterations related to chronic inflammation can provide an environment conducive to prohypertensive inflammation [113]. There are data suggesting that moderate to severe periodontal disease is associated with a higher likelihood of hypertension. Figure 3 illustrates the systemic pathologies associated with periodontal disease [78,114,115].

### 5.1. Periodontal Disease and COVID-19 Severity

Some comorbidities such as diabetes mellitus, obesity, and heart disease have been shown to play an important role in determining the prognosis of COVID-19 [116]. It is also well established that periodontal disease has close links to these chronic conditions [117,118]. From the relationship between periodontal disease and COVID-19, the evidence suggested that the aspiration of periodontal pathogens may contribute to the aggravation of SARS-CoV-2 infection in the lungs by aggravating the secretion of key inflammatory cytokines, such as IL-6 [119]. Since saliva is an important route of transmission of SARS-CoV-2 infection, saliva droplets may contain a high viral load, especially during the early stages of infection [120]. This could contribute to the spread of SARS-CoV-2 when infected individuals cough or sneeze, but in addition, inhalation of saliva droplets can transport SARS-CoV-2 to the lower respiratory tract [121]. Considering that the infected mouth and periodontium with infection are important sources of respiratory pathogenic microorganisms, the risk of pulmonary infection increased in those individuals with poor oral hygiene and active periodontal infection [122,123]. With the fact that the prevalence of *P. gingivalis* infection increases with age, this periodontal pathogen has been implicated as one of the etiological factors in aspiration pneumonia in elderly patients, in the same way as in acute infection of both lungs [124,125]. Knowing that aspiration pneumonia is frequently caused by *P. gingivalis* [126] and that it has been possible to identify oral bacteria in bronchoalveolar lavage samples from COVID-19 [127].

Recent research has confirmed that the risk of several adverse events in patients with COVID-19 is considerably higher in patients with periodontitis [128,129]; this resulted from a multivariate logistic regression model in which they found that the associations between patients who had COVID-19 and patients who had periodontitis adjusted for possible confounders such as gender, age, and smoking, as well as comorbidities they might suffer from; after this adjustment, periodontitis undoubtedly had a highly significant impact on the progression and evolution of SARS-CoV-2 infection, having also highly significant associations with health complications caused by COVID-19 (OR = 3.67, 95% CI: 1.46–9.27), ICU admission (OR = 3.54, 95% CI: 1.39–9.05), death (OR = 8.81, 95% CI: 1.00–77.7), and need for mechanical ventilation (OR = 4.57, 95% CI: 1.19–17.4) when compared to patients with mild periodontal disease or healthy periodontium [14]. Periodontal disease shares very similar risk factors with many of the chronic inflammatory diseases, which have been reported to directly influence the severity of COVID-19 [128]; as already described, periodontitis has a significant effect and impact on the progression of COVID-19 infection and a significantly important risk with complications that can be severe in patients with COVID-19, death, admission to the intensive care unit, and the need for assisted ventilation, confirming the association between periodontal disease and a worse evolution of COVID-19 [14].

The observed association between periodontal disease and COVID-19 severity indicates that aspiration of bacteria involved in periodontal pathology could aggravate COVID-19 by inducing the expression of inflammatory cytokines and ACE2 in the lower respiratory tract [130]. It has also been suggested that bacteria involved in periodontal disease may increase the virulence of SARS-CoV-2 by cleaving the S-glycoprotein [130,131] and that in the mouth, the periodontal pockets, in particular, act as a natural reservoir for viruses [132,133,134,135,136]. Helper 17 lymphocyte response in severe periodontitis may aggravate the cytokine storm in COVID-19 [137], which could predict an increase in the incidence of periodontal lesions, in particular necrotizing periodontitis in the course of the current COVID-19 pandemic [50].

### 5.2. Periodontal Findings after COVID-19

Several types of enanthem have been described, such as aphthous ulcers, Koplik’s spots, Nagayama’s spots, petechiae, papulovesicular or maculopapular lesions, white or red spots, gingival and labial swelling, due to various viral infections [52]. Mucous membranes with keratinocytes (anterior palate, various types of gingiva, and tongue) and those without keratinocytes (labial and buccal mucosa) are usually affected [138]. It has been shown that 7% of patients with a positive reverse transcription polymerase chain reaction (RT-PCR) test for SARS-CoV-2 have plaque-like changes on the dorsum of the tongue. In addition, inflammation of the oral cavity (including lingual and gingival palate) was reported by 8% of patients. In addition, the occurrence of oral lesions was accompanied by loss of taste and smell in patients, and the most severe oral lesions tended to spread in older patients and mainly in the most severe cases of COVID-19 [43]. In another investigation, the presence of enanthem was also reported in 29% of cases with confirmed COVID-19 in addition to cutaneous exanthema [48]. One study found that patients with moderate cases of COVID-19 infection had an association with the presence of oral symptoms, so greater importance should be given to dental examination; they also observed that the incidence of oral manifestations in relation to oral hygiene measures taken by patients while infected with COVID-19 showed a statistically significant difference [60]. If we compare the presence of oral lesions in children infected with SARS-CoV-2 with those in adults, they are milder, in terms of cough, shortness of breath, oxygen saturation, fatigue, and abdominal pain [139]. A study in pediatric patients reported the following oral lesions: hyperemic pharynx, oral pseudomembranous candidiasis, geographic tongue, and saburral tongue, with herpetic pharynx being the most common [61].

## 6. Pharmacological Therapy for the Treatment of Oral Lesions in Patients with COVID-19

The use of drugs for the treatment of some oral lesions has been reported, including topical agents such as 2% chlorhexidine for lacerations, hyaluronic acid, tranexamic acid for local bleeding, and miconazole for patients with a cytological diagnosis of candidiasis [57]. Cases have been described of patients with erythema multiforme-like lesions using topical antiseptic medications of 0.2% chlorhexidine solution, in addition to the prescription of vitamin C, B complex, systemic corticosteroids, and panthenol-calcium with pantothenic acid (locally applied tablets); promotion of healing of the labial mucosa in these patients was performed with the typical application of Vaseline [48,140].

Angular cheilitis in most cases has been treated with nystatin, neomycin, and triamcinolone acetonide 0.05%. Xerostomia has been treated with artificial saliva (gels); it has been reported that for burning sensation in the oral cavity the indicated treatment has been triamcinolone acetonide 0.05%, as well as for aphthous lesions [44,54]. In cases of coinfection by herpes simplex virus, systemic acyclovir and some local antiseptics such as panthenol or nystatin have been indicated [73], and if there is also coinfection with candidiasis, intravenous fluconazole, mouthwash with 0.12% chlorhexidine digluconate, mouthwash with 1% hydrogen peroxide, and nystatin have been applied, as well as white plates [62]. Chlorhexidine 0.12% (mouthwash), hyaluronic acid, and prednisolone have been indicated to counteract oral blisters [68]. It has been observed that oral ulcers have healed completely within 10 days after their appearance [141]. When facial nerve paralysis is present, favipiravir and ciclesonide have been reported to improve facial nerve paralysis [142].

Is important to note that many of the pharmacotherapeutics agents used in COVID-19 are also used to treat oral diseases, especially orofacial pain and inflammatory conditions. However, analgesics (paracetamol, nonsteroidal anti-inflammatory agents), antivirals (penciclovir), antibiotics (azithromycin, doxycycline), and immunomodulatory agents (hydroxychloroquine, corticosteroids), have been associated with long-term adverse effects that complicate dental treatment [143]. Treatments involving more than two drugs and invasive therapeutics may cause immune function to be diminished or affected, thus worsening oral conditions, especially when candidiasis is present in the oropharynx [144].

A risk factor for negative outcomes in patients with COVID-19 and associated fungal infection is prolonged antibiotic use in association with immune dysregulation [144]. Perioral pressure ulcers, oral candidiasis, herpetic and hemorrhagic oral ulcer, and acute macroglia were the most commonly complicated health conditions in those patients who presented severe COVID-19 [145]. These oral mucocutaneous conditions were triggered by prolonged prone position and mechanical ventilation equipment in the intensive care unit (ICU) setting, along with the immunosuppressive treatments prescribed to these patients [145].

## 7. Vaccination and Mucosal Immunity

Mucosal immunity is mainly constituted by tissues, nonlymphoid cells, lymphocytes, and effector molecules [146]. Injectable vaccines have been described to be deficient inducers of mucosal immunity, whereas intranasal immunization induces important mucosal immunity to prevent pathogen entry, when SARS-CoV-2 enters the nasal cavity, the first barrier against viral infection is the respiratory epithelial layer [147]. After this, the innate immunity in the upper airway mucosa becomes the first line of defense, consisting of various immune cells, including phagocytic neutrophils, macrophages, dendritic cells, natural killer cells, and mast cells [148], such immune cells form an integrated system that fights against pathogens or induce adaptive mucosal immunity [149]. In situations where viruses reach the deeper airways or lungs, pathogen-associated molecular patterns expressed on viruses will be recognized by pattern recognition receptors expressed on respiratory epithelial cells, dendritic cells, and macrophages located in lung alveoli [150,151]. Surrounding viral particles are degraded by endosomal enzymes, which release single-stranded RNA that can be recognized by toll-like receptor (TLR) 7 or TLR8 [152]. Activation of pattern recognition receptors induces a significantly elevated expression of proinflammatory cytokines such as type I interferon (IFN-I) and IFN-III, which are important for fighting viral infection; however, SARS-CoV-2 infection induces a down-regulated expression of IFN-I and IFN-III [153,154].

Mucosal immunization induces extensive adaptive immune responses, characterized by resident memory T cells (T _RM_) and mucosal secretory IgA antibodies (sIgA) [155]. sIgA antibodies neutralize toxins or pathogens in the mucosa through three pathways: immune exclusion, intracellular neutralization, and antigen excretion [156,157]. Mucosal sIgA levels are known to increase rapidly in infants and reach adult levels in early childhood, while serum IgA levels increase more slowly and may not reach adult levels until puberty [158]. These presumed differences in the susceptibility of children and adults to COVID-19 must be taken into account in the SARS-CoV-2 vaccine development process [159].

The most effective method to combat the pandemic caused by SARS-CoV-2 is vaccination; currently, all vaccines that have been approved by the World Health Organization to combat SARS-CoV-2 are administered parenterally, inducing high titers of systemic neutralizing antibodies that can neutralize systemic viral infections [160]. Mucosal vaccines are administered by oral, vaginal, intranasal, ocular, rectal, or sublingual routes, although oral and intranasal routes of administration are the most commonly used. Oral immunization can induce strong immune responses in the mammary and salivary glands, as well as in the gastrointestinal tract [161]. The roles of mucous membranes in SARS-CoV-2 transmission and disease progression must be fully understood, as this helps to support the benefits of mucosal immunization with oral or intranasal vaccines [150]. Previously successful mucosal vaccines used to prevent infectious diseases have been effective in inducing and activating the mucosal immune system [162].

Some optimists have already been reported; one of them is the use of different routes of administration in subsequent doses of vaccination, both by the intramuscular and intranasal routes. This application in rhesus macaques showed efficient immune responses against SARS-CoV-2 infection [163]. In a golden hamster animal model, in which the pathophysiology of COVID-19 is very similar to that observed in humans, immunization with intranasal vaccine showed a significant prophylactic effect, which helped prevent excessive lung injury [164]. Another intranasal subunit vaccine containing trimeric or monomeric protein S combined with adjuvant induced strong mucosal immunity in mice [165].

One study reported the generation of a vaccine that, with a single intranasal dose administered to mice, caused a significant T and B cell-mediated immune response and induced IgA antibodies in the mucosa, resulting in complete protection of the animals against SARS-CoV-2 infection [166]. The SARS-CoV-2 structural protein N has received attention as a potential vaccine target. In one study, intranasal vaccination of BALB/c mice with a recombinant adenovirus type 5 (Ad5) expressing the SARS-CoV-2 N protein induced significant amounts of CD8+ and CD4+ T-cell-mediated immune responses [167].

## 8. COVID-19: Dental Care and Staff Biosecurity

Dentistry is the profession that deals with the health of the oral cavity in humans, this cavity is undoubtedly the main route of transmission of COVID-19 (small droplets of saliva when coughing or sneezing) [168]; with dental staff and dental offices becoming a focus for the spread of infection, from patients to dentists and from patients to patients, it is important that dentists and dental practices keep up to date with all aspects of COVID-19 [53,169,170]. Adequate guidelines are now in place to protect dentists against SARS-CoV-2 during dental practice and the use of aerosols, although there are guidelines that do not fully prevent transmission caused by aerosols [169,170]. Some statistics show that 0.9% of the surveyed dentists contracted COVID-19 infection, which may indicate that social distancing is an appropriate measure in consultation with dentists to control the spread of SARS-CoV-2 [171]. Although thorough disinfection with chlorine or alcohol-based solutions can inactivate the virus on surfaces, reports indicate that SARS-CoV-2 survives on some surfaces for up to 9 days [172] and is estimated to have an average life of approximately 6–8 h in plastic and 5–6 h in stainless steel [173]. The persistence of SARS-CoV-2 was also detected in surgical masks, where the virus was observed to persist for up to 7 days [174]. Adequate hand washing is essential to combat and control the spread of many diseases [175]. Proper hand washing should be performed for at least 60 s to remove infectious microorganisms, especially if combined with hydroalcoholic solutions that aid in the inactivation of enveloped viruses, including coronaviruses [176]; adequate provision of personal protective equipment is the first and foremost requirement to ensure that healthcare personnel [177]. For proper dental treatment, most dentists prescribed a mouth rinse before starting any procedure. The rinses they recommended using were hydrogen peroxide or chlorhexidine [178,179,180], since the presence of viruses in saliva represents an important risk factor [181,182]. During dental practice, aerosol production should be limited [183,184], limiting the use of handpieces, the use of surgical suction, the four-handed technique, ventilation, and disinfection of surfaces are effective in preventing infections [173,185]. It is considered necessary to consider the recommendations listed in Table 2 for dental care.

## 9. Conclusions

The findings on oral involvement in COVID-19 may be extensive, since oral mucosal epithelial cells and salivary glands express ACE2, which, as already described in this literature review, is the entry receptor protein for SARS-CoV-2 in host cells [199]. This indicates that the oral cavity is an accessible and privileged environment for SARS-CoV-2 interaction with the mucosal immune system and target cells [62]; direct effects of the virus in this cavity worsen the antiviral inflammatory response of underlying oral disorders, in particular those related to immune deficiencies and autoimmunity [200]. Oral symptomatology in patients with COVID-19 is infrequently described in most of the clinical reports of the disease. There remains uncertainty about the evidence described for oral mucosal lesions in SARS-CoV-2-infected patients, and there is still insufficient information about their etiopathogenesis. Loss of taste and/or smell has been reported to be sustained for up to 14 days and progresses more rapidly in older patients; recovery from oral lesions occurs at the same time as patients recover from COVID-19, correlating the occurrence of oral lesions with SARS-CoV-2 infection [10]. In addition to the loss of taste and loss of smell experienced by patients with COVID-19, the oral manifestations reported are very varied, ranging from inflammation of Wharton’s duct to inflammation of the tongue papillae, tongue depapilation, xerostomia, candida-associated lesions, recurrent herpesvirus infection, aphthous lesions, ulcers, erythema-multiforme-type lesions, necrotizing periodontitis, and salivary gland infections [10,37,44,50,69,201,202,203,204,205]. Based on what has been reported and including the clinical aspects presented by the patients, it has been suggested that coinfections, immunosuppression, and poor oral hygiene are related to COVID-19 in the search for oral mucosal lesions caused by SARS-CoV-2 infection. Because this review article shows several periodontal and buccal mucosal lesions that are likely to be associated with COVID-19, we observed that some properly designed prospective cohort or case–control studies are essential to determine the relationship between COVID-19 and the etiology of oral lesions. If such studies were conducted, they could be carried out in several centers or hospitals, which would facilitate the collection of a larger number of patients. The veracity and validity of the diagnoses provided in relation to oral lesions should be considered, in addition to standardizing them according to their type and location. There could also be reports indicating the treatment provided to patients with COVID-19 to heal the oral lesions related to SARS-CoV-2 infection.

## Figures and Tables

**Table 1:** Representative studies evaluating oral manifestations in patients with COVID-19.

Design/Reference	Oral Lesions (Percentage, Frequency)	Sample	Country
Case series, Sinadinos, 2020 [9]	Herpetic recurrent stomatitis (n = 2, 67%) Erythema multiform (n = 1, 33%)	3	UK
Cross-sectional study, Mascitti, 2020 [52]	Oral lichenoid lesions (n = 13, 32.5%) Oral enanthem (n = 11, 27.5%) Macroglossia (n = 10, 25%) Herpes (n = 1, 2.5%) Cheilitis (n = 5, 12.5%)	40	France
Case series, Carreras-Presas, 2021 [53]	Herpetic recurrent stomatitis (n = 1, 33.3%) Multiple ulcerations on palate (n = 1, 33.3%) Desquamative gingivitis (n = 1, 33.3%)	3	Spain
Cross-sectional study Katz, 2021 [54]	Aphthous stomatitis (n = 6, 0.67%)	889	USA
Cross-sectional study Halepa, 2021 [55]	Red or swollen lips (n = 23, 48.9%) Strawberry tongue = (n = 5, 10.6%) Other oral manifestation (n = 7, 14.9%)	47	USA
Cross-sectional study Fidan, 2021 [56]	Aphthous-like ulcers (n = 27, 46.6%) Erythema (n = 19, 32.8%) Lichen planus (n = 12, 20.6%)	74	Turkey
Cross-sectional study Favia, 2021 [57]	Geographic tongue (n = 2, 2%) Fissured tongue (n = 3, 2%) Ulcerative lesion (n = 65, 53%) Blisters (n = 19, 15%) Hyperplasia of papillae (n = 48, 39%) Angina bullosa (n = 11, 9%) Candidiasis (n = 28, 23%) Necrotizing ulcerative gingivitis (n = 7, 6%) Petechiae (n = 14, 11%) Spontaneous oral hemorrhage (n = 1, 1%)	123	Italy
Cross-sectional study Falah, 2020 [58]	Oral cavity changes (n = 9, 90%) (unspecified)	10	Pakistan
Case series Cruz Tapia, 2020 [59]	Angina bullosa hemorrhagic-like lesion (n = 2, 50%) Vascular disorder associated with COVID-19 (n = 1, 25%) Mucosal nonspecific localized vasculitis and thrombosis (n = 1, 25%)	4	Columbia, Brazil, and Mexico
Case series Brandão, 2021 [10]	Ulcer (n = 4, 50%) Focal erythema/petechiae (n = 1, 13%) Aphthous-like ulcers (n = 2, 25%) Hemorrhagic ulcer (n = 2, 25%)	5	Brazil
Cross-sectional study Abubakr, 2021 [60]	Ulcerations (n = 117, 20.4%) Xerostomia (n = 273, 47.6%) Oral or dental pain (n = 132, 23%) Pain in jaw joint (n = 69, 12%) Halitosis (n = 60, 10.5%)	665	Egypt
Cross-sectional study Retrospective study Bardellini, 2021 [61]	Oral pseudomembranous candidiasis (n = 2, 7.4%) Geographic tongue (n = 1, 3.7%) Coated tongue (n = 2, 7.4%)	27	Italy

**Table 2:** Recommendations and procedures that should be performed during dental care throughout the COVID-19 pandemic.

Priority Level Consideration	Description/Recommendation
Priority cases for dental health care	Pericoronitis, pulpitis, abscess, osteitis, localized bacterial infection, dental trauma, extensive caries, broken or defective restorations causing tissue damage or pain, suture removal, adjustments to appliances and prostheses causing damage to oral structures, and replacement of temporary endodontic fillings and patients with presence of pain [186].
Nonpriority dental procedures	Cosmetic dental procedures, orthodontic and orthopedic treatments, replacement of permanent restorations for aesthetic reasons, intentional root canal treatment, elective periodontal care, and nonurgent oral surgery and prosthetics [186].
Regarding location of dental care personnel	It is of vital importance to avoid overcrowding in all areas of circulation of patients and health personnel in addition to waiting rooms; take a distance of not less than 1.8 m within the room or care area of the cases that have been confirmed with COVID-19 [187].
Appointments and schedules	Appointing patients at established times, which must be respected, with a safe time interval between each of the dental consultations, avoiding contact and approach between patients [187].
During the emergency dental consultation	- Evaluate patients suspected of COVID-19 by signs and symptoms, particularly cough and fever [188]. - Attenuate the symptoms that provoke pain and cause inflammation in the dental organs, this can be with analgesics and anti-inflammatory drugs [189]. - Provide information to staff and provide them with the use of personal protective equipment (N95 respirators, safety goggles, clinical gloves, disposable aprons, and hair covers) [190,191]. - Wash hands with soap and water or clean them with 70% isopropanol or 70% ethanol before and after care [186]. - Do not touch the nose, mouth, and eyes without having cleaned hands [192,193]. - Use computed tomography and extraoral radiographs in preference to intraoral radiographs, and avoid the generation of lumps in the areas that trigger coughing and vomiting [194,195]. - Use rubber dam for absolute isolation during the dental procedure and do not use air-water syringe lavage, ultrasonic and sonic scalers, rotary handpieces, and air abrasion units [196]. - Do not remove the mask before 30 min in the environment where dental care is performed because it increases the risk of contagion [197]. - Disinfect all surfaces and structures of the dental office and patient care equipment with hospital germicides. Proper sterilization of all dental instruments should be performed according to the biosafety techniques that have been established by the manufacturers of the devices used for sterilization [173]. - Dispose of the remains of all dental procedures in special packages or bags for infected waste [198].

## Data Availability

Not applicable.

## References

[1] Chaimayo C., Kaewnaphan B., Tanlieng N., Athipanyasilp N., Sirijatuphat R., Chayakulkeeree M., Angkasekwinai N., Sutthent R., Puangpunngam N., Tharmviboonsri T. (2020). Rapid SARS-CoV-2 antigen detection assay in comparison with real-time RT-PCR assay for laboratory diagnosis of COVID-19 in Thailand. Virol. J..

[2] Zhu F.C., Li Y.H., Guan X.H., Hou L.H., Wang W.J., Li J.X., Wu S.P., Wang B.S., Wang Z., Wang L. (2020). Safety, tolerability, and immunogenicity of a recombinant adenovirus type-5 vectored COVID-19 vaccine: A dose-escalation, open-label, non-randomised, first-in-human trial. Lancet.

[3] Hoffmann M., Kleine-Weber H., Schroeder S., Kruger N., Herrler T., Erichsen S., Schiergens T.S., Herrler G., Wu N.H., Nitsche A. (2020). SARS-CoV-2 Cell Entry Depends on ACE2 and TMPRSS2 and Is Blocked by a Clinically Proven Protease Inhibitor. Cell.

[4] Galvan Casas C., Catala A., Carretero Hernandez G., Rodriguez-Jimenez P., Fernandez-Nieto D., Rodriguez-Villa Lario A., Navarro Fernandez I., Ruiz-Villaverde R., Falkenhain-Lopez D., Llamas Velasco M. (2020). Classification of the cutaneous manifestations of COVID-19: A rapid prospective nationwide consensus study in Spain with 375 cases. Br. J. Dermatol..

[5] Al-Khatib A. (2021). Oral manifestations in COVID-19 patients. Oral Dis..

[6] Dinnes J., Deeks J.J., Berhane S., Taylor M., Adriano A., Davenport C., Dittrich S., Emperador D., Takwoingi Y., Cunningham J. (2021). Rapid, point-of-care antigen and molecular-based tests for diagnosis of SARS-CoV-2 infection. Cochrane Database Syst. Rev..

[7] Bemquerer L.M., de Arruda J.A.A., Soares M.P.D., Mesquita R.A., Silva T.A. (2021). The oral cavity cannot be forgotten in the COVID-19 era: Is there a connection between dermatologic and oral manifestations?. J. Am. Acad. Dermatol..

[8] Amorim Dos Santos J., Normando A.G.C., Carvalho da Silva R.L., Acevedo A.C., De Luca Canto G., Sugaya N., Santos-Silva A.R., Guerra E.N.S. (2021). Oral Manifestations in Patients with COVID-19: A 6-Month Update. J. Dent. Res..

[9] Sinadinos A., Shelswell J. (2020). Oral ulceration and blistering in patients with COVID-19. Evid. Based Dent..

[10] Brandao T.B., Gueiros L.A., Melo T.S., Prado-Ribeiro A.C., Nesrallah A., Prado G.V.B., Santos-Silva A.R., Migliorati C.A. (2021). Oral lesions in patients with SARS-CoV-2 infection: Could the oral cavity be a target organ?. Oral Surg. Oral Med. Oral Pathol. Oral Radiol..

[11] Kamel A.H.M., Basuoni A., Salem Z.A., AbuBakr N. (2021). The impact of oral health status on COVID-19 severity, recovery period and C-reactive protein values. Br. Dent. J..

[12] Katz J., Yue S., Xue W. (2022). Dental diseases are associated with increased odds ratio for coronavirus disease 19. Oral Dis..

[13] Larvin H., Wilmott S., Wu J., Kang J. (2020). The Impact of Periodontal Disease on Hospital Admission and Mortality During COVID-19 Pandemic. Front. Med..

[14] Marouf N., Cai W., Said K.N., Daas H., Diab H., Chinta V.R., Hssain A.A., Nicolau B., Sanz M., Tamimi F. (2021). Association between periodontitis and severity of COVID-19 infection: A case-control study. J. Clin. Periodontol..

[15] Fabian T.K., Hermann P., Beck A., Fejerdy P., Fabian G. (2012). Salivary defense proteins: Their network and role in innate and acquired oral immunity. Int. J. Mol. Sci..

[16] Diamond G., Beckloff N., Ryan L.K. (2008). Host defense peptides in the oral cavity and the lung: Similarities and differences. J. Dent. Res..

[17] Walker D.M. (2004). Oral mucosal immunology: An overview. Ann. Acad. Med. Singap..

[18] Pivarcsi A., Bodai L., Rethi B., Kenderessy-Szabo A., Koreck A., Szell M., Beer Z., Bata-Csorgoo Z., Magocsi M., Rajnavolgyi E. (2003). Expression and function of Toll-like receptors 2 and 4 in human keratinocytes. Int. Immunol..

[19] Cook D.N., Pisetsky D.S., Schwartz D.A. (2004). Toll-like receptors in the pathogenesis of human disease. Nat. Immunol..

[20] Palm N.W., Medzhitov R. (2009). Pattern recognition receptors and control of adaptive immunity. Immunol. Rev..

[21] de Koning H.D., Rodijk-Olthuis D., van Vlijmen-Willems I.M., Joosten L.A., Netea M.G., Schalkwijk J., Zeeuwen P.L. (2010). A comprehensive analysis of pattern recognition receptors in normal and inflamed human epidermis: Upregulation of dectin-1 in psoriasis. J. Investig. Dermatol..

[22] Hoebe K., Janssen E., Beutler B. (2004). The interface between innate and adaptive immunity. Nat. Immunol..

[23] DePaolo R.W., Kamdar K., Khakpour S., Sugiura Y., Wang W., Jabri B. (2012). A specific role for TLR1 in protective T(H)17 immunity during mucosal infection. J. Exp. Med..

[24] Blander J.M., Sander L.E. (2012). Beyond pattern recognition: Five immune checkpoints for scaling the microbial threat. Nat. Rev. Immunol..

[25] Haynes B.F., Soderberg K.A., Fauci A.S., Kasper D., Fauci A., Hauser S., Longo D., Jameson J.L., Loscalzo J. (2014). Introduction to the Immune System. Harrison’s Principles of Internal Medicine.

[26] Kouketsu A., Sato I., Abe S., Oikawa M., Shimizu Y., Takahashi T., Kumamoto H. (2016). Detection of human papillomavirus infection in oral squamous cell carcinoma: A cohort study of Japanese patients. J. Oral Pathol. Med..

[27] Nayyar S.S., Thiagarajan S., Malik A., D’Cruz A., Chaukar D., Patil P., Alahari A.D., Lashkar S.G., Prabhash K. (2020). Head and neck squamous cell carcinoma in HIV, HBV and HCV seropositive patients—Prognosis and its predictors. J. Cancer Res. Ther..

[28] Ottria L., Lauritano D., Oberti L., Candotto V., Cura F., Tagliabue A., Tettamanti L. (2018). Prevalence of HIV-related oral manifestations and their association with HAART and CD4+ T cell count: A review. J. Biol. Regul. Homeost. Agents.

[29] Ceballos-Salobrena A., Gaitan-Cepeda L.A., Ceballos-Garcia L., Lezama-Del Valle D. (2000). Oral lesions in HIV/AIDS patients undergoing highly active antiretroviral treatment including protease inhibitors: A new face of oral AIDS?. AIDS Patient Care STDs.

[30] Falschlehner C., Schaefer U., Walczak H. (2009). Following TRAIL’s path in the immune system. Immunology.

[31] Gabay C., Kushner I. (1999). Acute-phase proteins and other systemic responses to inflammation. New Engl. J. Med..

[32] Oved K., Cohen A., Boico O., Navon R., Friedman T., Etshtein L., Kriger O., Bamberger E., Fonar Y., Yacobov R. (2015). A novel host-proteome signature for distinguishing between acute bacterial and viral infections. PLoS ONE.

[33] Scully C., Diz Dios P. (2001). Orofacial effects of antiretroviral therapies. Oral Dis..

[34] Ruiz-Garbajosa P., Canton R. (2021). COVID-19: Impact on prescribing and antimicrobial resistance. Rev. Esp. Quimioter..

[35] Gloster A.T., Lamnisos D., Lubenko J., Presti G., Squatrito V., Constantinou M., Nicolaou C., Papacostas S., Aydin G., Chong Y.Y. (2020). Impact of COVID-19 pandemic on mental health: An international study. PLoS ONE.

[36] Bajaj N., Granwehr B.P., Hanna E.Y., Chambers M.S. (2020). Salivary detection of SARS-CoV-2 (COVID-19) and implications for oral health-care providers. Head Neck.

[37] Sakaguchi W., Kubota N., Shimizu T., Saruta J., Fuchida S., Kawata A., Yamamoto Y., Sugimoto M., Yakeishi M., Tsukinoki K. (2020). Existence of SARS-CoV-2 Entry Molecules in the Oral Cavity. Int. J. Mol. Sci..

[38] Jermy M.C., Spence C.J.T., Kirton R., O’Donnell J.F., Kabaliuk N., Gaw S., Hockey H., Jiang Y., Zulkhairi Abidin Z., Dougherty R.L. (2021). Assessment of dispersion of airborne particles of oral/nasal fluid by high flow nasal cannula therapy. PLoS ONE.

[39] Huang N., Perez P., Kato T., Mikami Y., Okuda K., Gilmore R.C., Conde C.D., Gasmi B., Stein S., Beach M. (2021). SARS-CoV-2 infection of the oral cavity and saliva. Nat. Med..

[40] Vieira Braga F.A., Kar G., Berg M., Carpaij O.A., Polanski K., Simon L.M., Brouwer S., Gomes T., Hesse L., Jiang J. (2019). A cellular census of human lungs identifies novel cell states in health and in asthma. Nat. Med..

[41] Vaira L.A., Hopkins C., Salzano G., Petrocelli M., Melis A., Cucurullo M., Ferrari M., Gagliardini L., Pipolo C., Deiana G. (2020). Olfactory and gustatory function impairment in COVID-19 patients: Italian objective multicenter-study. Head Neck.

[42] Lechien J.R., Chiesa-Estomba C.M., De Siati D.R., Horoi M., Le Bon S.D., Rodriguez A., Dequanter D., Blecic S., El Afia F., Distinguin L. (2020). Olfactory and gustatory dysfunctions as a clinical presentation of mild-to-moderate forms of the coronavirus disease (COVID-19): A multicenter European study. Eur. Arch. Otorhinolaryngol..

[43] Biadsee A., Kassem F., Dagan O., Masarwa S., Ormianer Z. (2020). Olfactory and Oral Manifestations of COVID-19: Sex-Related Symptoms-A Potential Pathway to Early Diagnosis. Otolaryngol.-Head Neck Surg..

[44] Diaz Rodriguez M., Jimenez Romera A., Villarroel M. (2020). Oral manifestations associated with COVID-19. Oral Dis..

[45] Tomo S., Miyahara G.I., Simonato L.E. (2020). Oral mucositis in a SARS-CoV-2-infected patient: Secondary or truly associated condition?. Oral Dis..

[46] Wang C., Wu H., Ding X., Ji H., Jiao P., Song H., Li S., Du H. (2020). Does infection of 2019 novel coronavirus cause acute and/or chronic sialadenitis?. Med. Hypotheses.

[47] Cebeci Kahraman F., Caskurlu H. (2020). Mucosal involvement in a COVID-19-positive patient: A case report. Dermatol. Ther..

[48] Jimenez-Cauhe J., Ortega-Quijano D., de Perosanz-Lobo D., Burgos-Blasco P., Vano-Galvan S., Fernandez-Guarino M., Fernandez-Nieto D. (2020). Enanthem in Patients With COVID-19 and Skin Rash. JAMA Dermatol..

[49] Corchuelo J., Ulloa F.C. (2020). Oral manifestations in a patient with a history of asymptomatic COVID-19: Case report. Int. J. Infect. Dis..

[50] Patel J., Woolley J. (2021). Necrotizing periodontal disease: Oral manifestation of COVID-19. Oral Dis..

[51] Aghazadeh N., Homayouni M., Sartori-Valinotti J.C. (2020). Oral vesicles and acral erythema: Report of a cutaneous manifestation of COVID-19. Int. J. Dermatol..

[52] Mascitti H., Bonsang B., Dinh A., Assan F., Perronne V., Leblanc T., Duran C., Bouchand F., Matt M., Le Gal A. (2020). Clinical Cutaneous Features of Patients Infected With SARS-CoV-2 Hospitalized for Pneumonia: A Cross-sectional Study. Open Forum. Infect Dis..

[53] Martin Carreras-Presas C., Amaro Sanchez J., Lopez-Sanchez A.F., Jane-Salas E., Somacarrera Perez M.L. (2021). Oral vesiculobullous lesions associated with SARS-CoV-2 infection. Oral Dis..

[54] Katz J., Yue S. (2021). Increased odds ratio for COVID-19 in patients with recurrent aphthous stomatitis. J. Oral Pathol. Med..

[55] Halepas S., Lee K.C., Myers A., Yoon R.K., Chung W., Peters S.M. (2021). Oral manifestations of COVID-2019-related multisystem inflammatory syndrome in children: A review of 47 pediatric patients. J. Am. Dent. Assoc..

[56] Fidan V., Koyuncu H., Akin O. (2021). Oral lesions in Covid 19 positive patients. Am. J. Otolaryngol..

[57] Favia G., Tempesta A., Barile G., Brienza N., Capodiferro S., Vestito M.C., Crudele L., Procacci V., Ingravallo G., Maiorano E. (2021). Covid-19 Symptomatic Patients with Oral Lesions: Clinical and Histopathological Study on 123 Cases of the University Hospital Policlinic of Bari with a Purpose of a New Classification. J. Clin. Med..

[58] Falah N.U., Hashmi S., Ahmed Z., Jaan A., Akhtar A., Khalid F., Farooque U., Shera M.T., Ali S., Javed A. (2020). Kawasaki Disease-Like Features in 10 Pediatric COVID-19 Cases: A Retrospective Study. Cureus.

[59] Cruz Tapia R.O., Peraza Labrador A.J., Guimaraes D.M., Matos Valdez L.H. (2020). Oral mucosal lesions in patients with SARS-CoV-2 infection. Report of four cases. Are they a true sign of COVID-19 disease?. Spec. Care Dent..

[60] Abubakr N., Salem Z.A., Kamel A.H.M. (2021). Oral manifestations in mild-to-moderate cases of COVID-19 viral infection in the adult population. Dent. Med. Probl..

[61] Bardellini E., Bondioni M.P., Amadori F., Veneri F., Lougaris V., Meini A., Plebani A., Majorana A. (2021). Non-specific oral and cutaneous manifestations of Coronavirus Disease 2019 in children. Med. Oral Patol. Oral Cir. Bucal..

[62] Amorim Dos Santos J., Normando A.G.C., Carvalho da Silva R.L., De Paula R.M., Cembranel A.C., Santos-Silva A.R., Guerra E.N.S. (2020). Oral mucosal lesions in a COVID-19 patient: New signs or secondary manifestations?. Int. J. Infect. Dis..

[63] Wu Y.H., Wu Y.C., Lang M.J., Lee Y.P., Jin Y.T., Chiang C.P. (2021). Review of oral ulcerative lesions in COVID-19 patients: A comprehensive study of 51 cases. J. Dent. Sci..

[64] Kammerer T., Walch J., Flaig M., French L.E. (2021). COVID-19-associated herpetic gingivostomatitis. Clin. Exp. Dermatol..

[65] Indu S. (2020). Multiple oral ulcerations—An initial manifestation of COVID 19 infection: A personal experience!!. J. Oral Maxillofac. Pathol..

[66] Soares C.D., Carvalho R.A., Carvalho K.A., Carvalho M.G., Almeida O.P. (2020). Letter to Editor: Oral lesions in a patient with Covid-19. Med. Oral Patol. Oral Y Cir. Bucal.

[67] Sakaida T., Tanimoto I., Matsubara A., Nakamura M., Morita A. (2020). Unique skin manifestations of COVID-19: Is drug eruption specific to COVID-19?. J. Dermatol. Sci..

[68] Ansari R., Gheitani M., Heidari F. (2021). Oral cavity lesions as a manifestation of the novel virus (COVID-19). Oral Dis..

[69] Jimenez-Cauhe J., Ortega-Quijano D., Carretero-Barrio I., Suarez-Valle A., Saceda-Corralo D., Moreno-Garcia Del Real C., Fernandez-Nieto D. (2020). Erythema multiforme-like eruption in patients with COVID-19 infection: Clinical and histological findings. Clin. Exp. Dermatol..

[70] Zangrillo A., Beretta L., Scandroglio A.M., Monti G., Fominskiy E., Colombo S., Morselli F., Belletti A., Silvani P., Crivellari M. (2020). Characteristics, treatment, outcomes and cause of death of invasively ventilated patients with COVID-19 ARDS in Milan, Italy. Crit. Care Resusc..

[71] Arens C., Herrmann I.F., Rohrbach S., Schwemmle C., Nawka T. (2015). Position paper of the German Society of Oto-Rhino-Laryngology, Head and Neck Surgery and the German Society of Phoniatrics and Pediatric Audiology-Current state of clinical and endoscopic diagnostics, evaluation, and therapy of swallowing disorders in children. GMS Curr. Top. Otorhinolaryngol. Head Neck Surg..

[72] Brakenhoff T.B., Franks B., Goodale B.M., van de Wijgert J., Montes S., Veen D., Fredslund E.K., Rispens T., Risch L., Dowling A.V. (2021). A prospective, randomized, single-blinded, crossover trial to investigate the effect of a wearable device in addition to a daily symptom diary for the remote early detection of SARS-CoV-2 infections (COVID-RED): A structured summary of a study protocol for a randomized controlled trial. Trials.

[73] Glavina A., Biocina-Lukenda D., Mravak-Stipetic M., Markeljevic J. (2020). Oral symptoms and lesions in SARS-CoV-2-positive patient. Oral Dis..

[74] Chousterman B.G., Swirski F.K., Weber G.F. (2017). Cytokine storm and sepsis disease pathogenesis. Semin. Immunopathol..

[75] Singh S., Sharma A., Arora S.K. (2014). High producer haplotype (CAG) of -863C/A, -308G/A and -238G/A polymorphisms in the promoter region of TNF-alpha gene associate with enhanced apoptosis of lymphocytes in HIV-1 subtype C infected individuals from North India. PLoS ONE.

[76] Liao Y.C., Liang W.G., Chen F.W., Hsu J.H., Yang J.J., Chang M.S. (2002). IL-19 induces production of IL-6 and TNF-alpha and results in cell apoptosis through TNF-alpha. J. Immunol..

[77] Aggarwal S., Gollapudi S., Gupta S. (1999). Increased TNF-alpha-induced apoptosis in lymphocytes from aged humans: Changes in TNF-alpha receptor expression and activation of caspases. J. Immunol..

[78] Tonetti M.S., Jepsen S., Jin L., Otomo-Corgel J. (2017). Impact of the global burden of periodontal diseases on health, nutrition and wellbeing of mankind: A call for global action. J. Clin. Periodontol..

[79] Eke P.I., Dye B.A., Wei L., Slade G.D., Thornton-Evans G.O., Borgnakke W.S., Taylor G.W., Page R.C., Beck J.D., Genco R.J. (2015). Update on Prevalence of Periodontitis in Adults in the United States: NHANES 2009 to 2012. J. Periodontol..

[80] Pan W., Wang Q., Chen Q. (2019). The cytokine network involved in the host immune response to periodontitis. Int. J. Oral Sci..

[81] Lacey D.C., Simmons P.J., Graves S.E., Hamilton J.A. (2009). Proinflammatory cytokines inhibit osteogenic differentiation from stem cells: Implications for bone repair during inflammation. Osteoarthr. Cartil..

[82] Wang H., Ai L., Zhang Y., Cheng J., Yu H., Li C., Zhang D., Pan Y., Lin L. (2018). The Effects of Antimicrobial Peptide Nal-P-113 on Inhibiting Periodontal Pathogens and Improving Periodontal Status. BioMed Res. Int..

[83] Balejo R.D.P., Cortelli J.R., Costa F.O., Cyrino R.M., Aquino D.R., Cogo-Muller K., Miranda T.B., Moura S.P., Cortelli S.C. (2017). Effects of chlorhexidine preprocedural rinse on bacteremia in periodontal patients: A randomized clinical trial. J. Appl. Oral Sci. Rev. FOB.

[84] Blum A. (2011). Dentistry and internal medicine: From the focal infection theory to the periodontal medicine concept-there could be light in the end of the tunnel. Eur. J. Intern. Med..

[85] Dominy S.S., Lynch C., Ermini F., Benedyk M., Marczyk A., Konradi A., Nguyen M., Haditsch U., Raha D., Griffin C. (2019). Porphyromonas gingivalis in Alzheimer’s disease brains: Evidence for disease causation and treatment with small-molecule inhibitors. Sci. Adv..

[86] Forner L., Larsen T., Kilian M., Holmstrup P. (2006). Incidence of bacteremia after chewing, tooth brushing and scaling in individuals with periodontal inflammation. J. Clin. Periodontol..

[87] Kinane D.F., Riggio M.P., Walker K.F., MacKenzie D., Shearer B. (2005). Bacteraemia following periodontal procedures. J. Clin. Periodontol..

[88] Lockhart P.B., Brennan M.T., Sasser H.C., Fox P.C., Paster B.J., Bahrani-Mougeot F.K. (2008). Bacteremia associated with toothbrushing and dental extraction. Circulation.

[89] Kozarov E.V., Dorn B.R., Shelburne C.E., Dunn W.A., Progulske-Fox A. (2005). Human atherosclerotic plaque contains viable invasive Actinobacillus actinomycetemcomitans and Porphyromonas gingivalis. Arterioscler. Thromb. Vasc. Biol..

[90] Belstrom D., Constancias F., Drautz-Moses D.I., Schuster S.C., Veleba M., Mahe F., Givskov M. (2021). Periodontitis associates with species-specific gene expression of the oral microbiota. NPJ Biofilms Microbiomes.

[91] Baelum V., Lopez R. (2013). Periodontal disease epidemiology-learned and unlearned?. Periodontology 2000.

[92] Monsarrat P., Blaizot A., Kemoun P., Ravaud P., Nabet C., Sixou M., Vergnes J.N. (2016). Clinical research activity in periodontal medicine: A systematic mapping of trial registers. J. Clin. Periodontol..

[93] Sanz M., Kornman K. (2013). Periodontitis and adverse pregnancy outcomes: Consensus report of the Joint EFP/AAP Workshop on Periodontitis and Systemic Diseases. J. Clin. Periodontol..

[94] Tonetti M.S., Van Dyke T.E. (2013). Periodontitis and atherosclerotic cardiovascular disease: Consensus report of the Joint EFP/AAPWorkshop on Periodontitis and Systemic Diseases. J. Periodontol..

[95] Couper D.J., Beck J.D., Falkner K.L., Graham S.P., Grossi S.G., Gunsolley J.C., Madden T., Maupome G., Offenbacher S., Stewart D.D. (2008). The Periodontitis and Vascular Events (PAVE) pilot study: Recruitment, retention, and community care controls. J. Periodontol..

[96] LaMonte M.J., Genco R.J., Hovey K.M., Wallace R.B., Freudenheim J.L., Michaud D.S., Mai X., Tinker L.F., Salazar C.R., Andrews C.A. (2017). History of Periodontitis Diagnosis and Edentulism as Predictors of Cardiovascular Disease, Stroke, and Mortality in Postmenopausal Women. J. Am. Heart Assoc..

[97] D’Aiuto F., Gkranias N., Bhowruth D., Khan T., Orlandi M., Suvan J., Masi S., Tsakos G., Hurel S., Hingorani A.D. (2018). Systemic effects of periodontitis treatment in patients with type 2 diabetes: A 12 month, single-centre, investigator-masked, randomised trial. Lancet. Diabetes Endocrinol..

[98] Suvan J.E., Petrie A., Nibali L., Darbar U., Rakmanee T., Donos N., D’Aiuto F. (2015). Association between overweight/obesity and increased risk of periodontitis. J. Clin. Periodontol..

[99] Tonetti M.S., D’Aiuto F., Nibali L., Donald A., Storry C., Parkar M., Suvan J., Hingorani A.D., Vallance P., Deanfield J. (2007). Treatment of periodontitis and endothelial function. N. Engl. J. Med..

[100] Sharma P., Dietrich T., Ferro C.J., Cockwell P., Chapple I.L. (2016). Association between periodontitis and mortality in stages 3-5 chronic kidney disease: NHANES III and linked mortality study. J. Clin. Periodontol..

[101] Chung J.H., Hwang H.J., Kim S.H., Kim T.H. (2016). Associations Between Periodontitis and Chronic Obstructive Pulmonary Disease: The 2010 to 2012 Korean National Health and Nutrition Examination Survey. J. Periodontol..

[102] Michaud D.S., Kelsey K.T., Papathanasiou E., Genco C.A., Giovannucci E. (2016). Periodontal disease and risk of all cancers among male never smokers: An updated analysis of the Health Professionals Follow-up Study. Ann. Oncol..

[103] Koshy B.S., Mahendra J. (2017). The Association between Periodontal Status, Serum Lipid Levels, Lipoprotein Associated Phosholipase A2 (Lp-PLA2) in Chronic Periodontitis Subjects and Healthy Controls. J. Clin. Diagn. Res..

[104] Dain C.P., Ganapathi S., Geevar Z., Harikrishnan S., Ammu J.V., Chacko M. (2021). The traditional and modifiable risk factors of coronary artery disease—A community-based cross-sectional study among 2 populations. Medicine.

[105] Shen T.C., Chang P.Y., Lin C.L., Chen C.H., Tu C.Y., Hsia T.C., Shih C.M., Hsu W.H., Sung F.C., Kao C.H. (2015). Risk of Periodontal Diseases in Patients With Chronic Obstructive Pulmonary Disease: A Nationwide Population-based Cohort Study. Medicine.

[106] Sanz M., Ceriello A., Buysschaert M., Chapple I., Demmer R.T., Graziani F., Herrera D., Jepsen S., Lione L., Madianos P. (2018). Scientific evidence on the links between periodontal diseases and diabetes: Consensus report and guidelines of the joint workshop on periodontal diseases and diabetes by the International diabetes Federation and the European Federation of Periodontology. Diabetes Res. Clin. Pract..

[107] Patel M.H., Kumar J.V., Moss M.E. (2013). Diabetes and tooth loss: An analysis of data from the National Health and Nutrition Examination Survey, 2003-2004. J. Am. Dent. Assoc..

[108] Grubbs V., Garcia F., Jue B.L., Vittinghoff E., Ryder M., Lovett D., Carrillo J., Offenbacher S., Ganz P., Bibbins-Domingo K. (2017). The Kidney and Periodontal Disease (KAPD) study: A pilot randomized controlled trial testing the effect of non-surgical periodontal therapy on chronic kidney disease. Contemp. Clin. Trials.

[109] Valenzuela-Narvaez R.V., Valenzuela-Narvaez D.R., Valenzuela-Narvaez D.A.O., Cordova-Noel M.E., Mejia-Ruiz C.L., Salcedo-Rodriguez M.N., Gonzales-Aedo O. (2021). Periodontal disease as a predictor of chronic kidney disease (CKD) stage in older adults. J. Int. Med. Res..

[110] Michaud D.S., Liu Y., Meyer M., Giovannucci E., Joshipura K. (2008). Periodontal disease, tooth loss, and cancer risk in male health professionals: A prospective cohort study. Lancet Oncol..

[111] Yao Y., Shen X., Zhou M., Tang B. (2021). Periodontal Pathogens Promote Oral Squamous Cell Carcinoma by Regulating ATR and NLRP3 Inflammasome. Front. Oncol..

[112] Tezal M., Sullivan M.A., Reid M.E., Marshall J.R., Hyland A., Loree T., Lillis C., Hauck L., Wactawski-Wende J., Scannapieco F.A. (2007). Chronic periodontitis and the risk of tongue cancer. Arch. Otolaryngol.-Head Neck Surg..

[113] Czesnikiewicz-Guzik M., Osmenda G., Siedlinski M., Nosalski R., Pelka P., Nowakowski D., Wilk G., Mikolajczyk T.P., Schramm-Luc A., Furtak A. (2019). Causal association between periodontitis and hypertension: Evidence from Mendelian randomization and a randomized controlled trial of non-surgical periodontal therapy. Eur. Heart J..

[114] Masi S., D’Aiuto F., Deanfield J. (2019). Cardiovascular prevention starts from your mouth. Eur. Heart J..

[115] Park S.Y., Kim S.H., Kang S.H., Yoon C.H., Lee H.J., Yun P.Y., Youn T.J., Chae I.H. (2019). Improved oral hygiene care attenuates the cardiovascular risk of oral health disease: A population-based study from Korea. Eur. Heart J..

[116] Zhang J., Wang X., Jia X., Li J., Hu K., Chen G., Wei J., Gong Z., Zhou C., Yu H. (2020). Risk factors for disease severity, unimprovement, and mortality in COVID-19 patients in Wuhan, China. Clin. Microbiol. Infect..

[117] Mei Y., Shen X., Wang X., Zhang M., Li Q., Yan J., Xu J., Xu Y. (2021). Expression of autophagy and apoptosis-related factors in the periodontal tissue of experimental diabetic rats: A histomorphometric, microtomographic and immunohistochemical study. PeerJ.

[118] Bobetsis Y.A., Kotsikoris I., Liapis C.D., Liasis N., Kakisis J., Kourlaba G., Lazari P., Antonopoulos C.N., Deliargyris E.N., Madianos P.N. (2020). Association between periodontal disease and vulnerable plaque morphology in patients undergoing carotid endarterectomy. Int. J. Cardiol. Heart Vasc..

[119] Hayata M., Watanabe N., Tamura M., Kamio N., Tanaka H., Nodomi K., Miya C., Nakayama E., Ueda K., Ogata Y. (2019). The Periodontopathic Bacterium Fusobacterium nucleatum Induced Proinflammatory Cytokine Production by Human Respiratory Epithelial Cell Lines and in the Lower Respiratory Organs in Mice. Cell. Physiol. Biochem..

[120] Yoon J.G., Yoon J., Song J.Y., Yoon S.Y., Lim C.S., Seong H., Noh J.Y., Cheong H.J., Kim W.J. (2020). Clinical Significance of a High SARS-CoV-2 Viral Load in the Saliva. J. Korean Med. Sci..

[121] Carrouel F., Goncalves L.S., Conte M.P., Campus G., Fisher J., Fraticelli L., Gadea-Deschamps E., Ottolenghi L., Bourgeois D. (2021). Antiviral Activity of Reagents in Mouth Rinses against SARS-CoV-2. J. Dent. Res..

[122] Javaheri N., Matin S., Naghizadeh-Baghi A., Bagheri A., Andreasian A., Ghobadi H. (2020). Periodontal Status, Its Treatment Needs, and Its Relationship with Airflow Limitation and Quality of Life in COPD Patients. Eurasian J. Med..

[123] Scannapieco F.A., Ho A.W. (2001). Potential associations between chronic respiratory disease and periodontal disease: Analysis of National Health and Nutrition Examination Survey III. J. Periodontol..

[124] Okuda K., Kimizuka R., Abe S., Kato T., Ishihara K. (2005). Involvement of periodontopathic anaerobes in aspiration pneumonia. J. Periodontol..

[125] Hajishengallis G., Wang M., Bagby G.J., Nelson S. (2008). Importance of TLR2 in early innate immune response to acute pulmonary infection with Porphyromonas gingivalis in mice. J. Immunol..

[126] Benedyk M., Mydel P.M., Delaleu N., Plaza K., Gawron K., Milewska A., Maresz K., Koziel J., Pyrc K., Potempa J. (2016). Gingipains: Critical Factors in the Development of Aspiration Pneumonia Caused by Porphyromonas gingivalis. J. Innate Immun..

[127] Shen Z., Xiao Y., Kang L., Ma W., Shi L., Zhang L., Zhou Z., Yang J., Zhong J., Yang D. (2020). Genomic Diversity of Severe Acute Respiratory Syndrome-Coronavirus 2 in Patients With Coronavirus Disease 2019. Clin. Infect. Dis..

[128] Ruan Q., Yang K., Wang W., Jiang L., Song J. (2020). Clinical predictors of mortality due to COVID-19 based on an analysis of data of 150 patients from Wuhan, China. Intensive Care Med..

[129] Zhou F., Yu T., Du R., Fan G., Liu Y., Liu Z., Xiang J., Wang Y., Song B., Gu X. (2020). Clinical course and risk factors for mortality of adult inpatients with COVID-19 in Wuhan, China: A retrospective cohort study. Lancet.

[130] Takahashi Y., Watanabe N., Kamio N., Kobayashi R., Iinuma T., Imai K. (2020). Aspiration of periodontopathic bacteria due to poor oral hygiene potentially contributes to the aggravation of COVID-19. J. Oral Sci..

[131] Madapusi Balaji T., Varadarajan S., Rao U.S.V., Raj A.T., Patil S., Arakeri G., Brennan P.A. (2020). Oral cancer and periodontal disease increase the risk of COVID 19? A mechanism mediated through furin and cathepsin overexpression. Med. Hypotheses.

[132] Badran Z., Gaudin A., Struillou X., Amador G., Soueidan A. (2020). Periodontal pockets: A potential reservoir for SARS-CoV-2?. Med. Hypotheses.

[133] Dela Cruz C.S., Wunderink R.G. (2017). Respiratory Viral and Atypical Pneumonias. Clin. Chest Med..

[134] Botros N., Iyer P., Ojcius D.M. (2020). Is there an association between oral health and severity of COVID-19 complications?. Biomed. J..

[135] Herrera D., Serrano J., Roldan S., Sanz M. (2020). Is the oral cavity relevant in SARS-CoV-2 pandemic?. Clin. Oral Investig..

[136] Kheur S., Kheur M., Gupta A.A., Raj A.T. (2020). Is the gingival sulcus a potential niche for SARS-Corona virus-2?. Med. Hypotheses.

[137] Sahni V., Gupta S. (2020). COVID-19 & Periodontitis: The cytokine connection. Med. Hypotheses.

[138] Rocha B.A., Souto G.R., Grossmann S.M.C., de Aguiar M.C.F., de Andrade B.A.B., Romanach M.J., Horta M.C.R. (2021). Viral enanthema in oral mucosa: A possible diagnostic challenge in the COVID-19 pandemic. Oral Dis..

[139] Li X., Xu S., Yu M., Wang K., Tao Y., Zhou Y., Shi J., Zhou M., Wu B., Yang Z. (2020). Risk factors for severity and mortality in adult COVID-19 inpatients in Wuhan. J. Allergy Clin. Immunol..

[140] Dalipi Z.S., Dragidella F., Dragidella D.K. (2021). Oral Manifestations of Exudative Erythema Multiforme in a Patient with COVID-19. Case Rep. Dent..

[141] Rusu L.C., Ardelean L.C., Tigmeanu C.V., Matichescu A., Sauciur I., Bratu E.A. (2021). COVID-19 and Its Repercussions on Oral Health: A Review. Medicina.

[142] Homma Y., Watanabe M., Inoue K., Moritaka T. (2020). Coronavirus Disease-19 Pneumonia with Facial Nerve Palsy and Olfactory Disturbance. Intern. Med..

[143] Dar-Odeh N., Elsayed S., Babkair H., Abu-Hammad S., Althagafi N., Bahabri R., Eldeen Y.S., Aljohani W., Abu-Hammad O. (2021). What the dental practitioner needs to know about pharmaco-therapeutic modalities of COVID-19 treatment: A review. J. Dent. Sci..

[144] Ohashi N., Ideta Y., Takeda A., Iwai T., Kioi M., Miyazaki A., Mitsudo K. (2021). Oral candidiasis caused by ciclesonide in a patient with COVID-19 pneumonia: A case report and literature review. SAGE Open Med. Case Rep..

[145] Hockova B., Riad A., Valky J., Sulajova Z., Stebel A., Slavik R., Beckova Z., Pokorna A., Klugarova J., Klugar M. (2021). Oral Complications of ICU Patients with COVID-19: Case-Series and Review of Two Hundred Ten Cases. J. Clin. Med..

[146] Arienti C., Pignatta S., Tesei A. (2019). Epidermal Growth Factor Receptor Family and its Role in Gastric Cancer. Front. Oncol..

[147] Villena J., Kitazawa H. (2020). The Modulation of Mucosal Antiviral Immunity by Immunobiotics: Could They Offer Any Benefit in the SARS-CoV-2 Pandemic?. Front. Physiol..

[148] Holmgren J., Czerkinsky C. (2005). Mucosal immunity and vaccines. Nat. Med..

[149] Yuan Q., Walker W.A. (2004). Innate immunity of the gut: Mucosal defense in health and disease. J. Pediatr. Gastroenterol. Nutr..

[150] Mudgal R., Nehul S., Tomar S. (2020). Prospects for mucosal vaccine: Shutting the door on SARS-CoV-2. Hum. Vaccin. Immunother..

[151] Moradi-Kalbolandi S., Majidzadeh A.K., Abdolvahab M.H., Jalili N., Farahmand L. (2021). The Role of Mucosal Immunity and Recombinant Probiotics in SARS-CoV2 Vaccine Development. Probiotics Antimicrob. Proteins.

[152] Akira S., Uematsu S., Takeuchi O. (2006). Pathogen recognition and innate immunity. Cell.

[153] Bowie A.G., Unterholzner L. (2008). Viral evasion and subversion of pattern-recognition receptor signalling. Nat. Rev. Immunol..

[154] Blanco-Melo D., Nilsson-Payant B.E., Liu W.C., Uhl S., Hoagland D., Moller R., Jordan T.X., Oishi K., Panis M., Sachs D. (2020). Imbalanced Host Response to SARS-CoV-2 Drives Development of COVID-19. Cell.

[155] Tacchi L., Musharrafieh R., Larragoite E.T., Crossey K., Erhardt E.B., Martin S.A.M., LaPatra S.E., Salinas I. (2014). Nasal immunity is an ancient arm of the mucosal immune system of vertebrates. Nat. Commun..

[156] Corthesy B. (2013). Multi-faceted functions of secretory IgA at mucosal surfaces. Front. Immunol..

[157] Rogier E.W., Frantz A.L., Bruno M.E., Kaetzel C.S. (2014). Secretory IgA is Concentrated in the Outer Layer of Colonic Mucus along with Gut Bacteria. Pathogens.

[158] Nurkic J., Numanovic F., Arnautalic L., Tihic N., Halilovic D., Jahic M. (2014). Diagnostic significance of reduced IgA in children. Med. Arch..

[159] Fischer A. (2020). Resistance of children to Covid-19. How?. Mucosal. Immunol..

[160] Kar S., Devnath P., Emran T.B., Tallei T.E., Mitra S., Dhama K. (2022). Oral and intranasal vaccines against SARS-CoV-2: Current progress, prospects, advantages, and challenges. Immun. Inflamm. Dis..

[161] Lycke N. (2012). Recent progress in mucosal vaccine development: Potential and limitations. Nat. Rev. Immunol..

[162] Vela Ramirez J.E., Sharpe L.A., Peppas N.A. (2017). Current state and challenges in developing oral vaccines. Adv. Drug Deliv. Rev..

[163] Sui Y., Li J., Zhang R., Prabhu S.K., Andersen H., Venzon D., Cook A., Brown R., Teow E., Velasco J. (2021). Protection against SARS-CoV-2 infection by a mucosal vaccine in rhesus macaques. JCI Insight.

[164] Ku M.W., Bourgine M., Authie P., Lopez J., Nemirov K., Moncoq F., Noirat A., Vesin B., Nevo F., Blanc C. (2021). Intranasal vaccination with a lentiviral vector protects against SARS-CoV-2 in preclinical animal models. Cell Host Microbe.

[165] An X., Martinez-Paniagua M., Rezvan A., Sefat S.R., Fathi M., Singh S., Biswas S., Pourpak M., Yee C., Liu X. (2021). Single-dose intranasal vaccination elicits systemic and mucosal immunity against SARS-CoV-2. iScience.

[166] Seo S.H., Jang Y. (2020). Cold-Adapted Live Attenuated SARS-Cov-2 Vaccine Completely Protects Human ACE2 Transgenic Mice from SARS-Cov-2 Infection. Vaccines.

[167] Swain S.L., McKinstry K.K., Strutt T.M. (2012). Expanding roles for CD4(+) T cells in immunity to viruses. Nat. Rev. Immunol..

[168] Huang C., Wang Y., Li X., Ren L., Zhao J., Hu Y., Zhang L., Fan G., Xu J., Gu X. (2020). Clinical features of patients infected with 2019 novel coronavirus in Wuhan, China. Lancet.

[169] Lo Giudice R. (2020). The Severe Acute Respiratory Syndrome Coronavirus-2 (SARS CoV-2) in Dentistry. Management of Biological Risk in Dental Practice. Int. J. Environ. Res. Public Health.

[170] Henrique Braz-Silva P., Pallos D., Giannecchini S., To K.K. (2021). SARS-CoV-2: What can saliva tell us?. Oral Dis..

[171] Estrich C.G., Mikkelsen M., Morrissey R., Geisinger M.L., Ioannidou E., Vujicic M., Araujo M.W.B. (2020). Estimating COVID-19 prevalence and infection control practices among US dentists. J. Am. Dent. Assoc..

[172] Dowell S.F., Simmerman J.M., Erdman D.D., Wu J.S., Chaovavanich A., Javadi M., Yang J.Y., Anderson L.J., Tong S., Ho M.S. (2004). Severe acute respiratory syndrome coronavirus on hospital surfaces. Clin. Infect. Dis..

[173] van Doremalen N., Bushmaker T., Morris D.H., Holbrook M.G., Gamble A., Williamson B.N., Tamin A., Harcourt J.L., Thornburg N.J., Gerber S.I. (2020). Aerosol and Surface Stability of SARS-CoV-2 as Compared with SARS-CoV-1. N. Engl. J. Med..

[174] Ong S.W.X., Tan Y.K., Chia P.Y., Lee T.H., Ng O.T., Wong M.S.Y., Marimuthu K. (2020). Air, Surface Environmental, and Personal Protective Equipment Contamination by Severe Acute Respiratory Syndrome Coronavirus 2 (SARS-CoV-2) From a Symptomatic Patient. JAMA.

[175] Goldberg J.L. (2017). Guideline Implementation: Hand Hygiene. AORN J..

[176] Lotfinejad N., Peters A., Pittet D. (2020). Hand hygiene and the novel coronavirus pandemic: The role of healthcare workers. J. Hosp. Infect..

[177] The Lancet (2020). COVID-19: Protecting health-care workers. Lancet.

[178] Carrouel F., Conte M.P., Fisher J., Goncalves L.S., Dussart C., Llodra J.C., Bourgeois D. (2020). COVID-19: A Recommendation to Examine the Effect of Mouthrinses with beta-Cyclodextrin Combined with Citrox in Preventing Infection and Progression. J. Clin. Med..

[179] Vergara-Buenaventura A., Castro-Ruiz C. (2020). Use of mouthwashes against COVID-19 in dentistry. Br J Oral Maxillofac Surg.

[180] Xu C., Wang A., Hoskin E.R., Cugini C., Markowitz K., Chang T.L., Fine D.H. (2021). Differential Effects of Antiseptic Mouth Rinses on SARS-CoV-2 Infectivity In Vitro. Pathogens.

[181] To K.K., Tsang O.T., Yip C.C., Chan K.H., Wu T.C., Chan J.M., Leung W.S., Chik T.S., Choi C.Y., Kandamby D.H. (2020). Consistent Detection of 2019 Novel Coronavirus in Saliva. Clin. Infect. Dis..

[182] To K.K., Tsang O.T., Leung W.S., Tam A.R., Wu T.C., Lung D.C., Yip C.C., Cai J.P., Chan J.M., Chik T.S. (2020). Temporal profiles of viral load in posterior oropharyngeal saliva samples and serum antibody responses during infection by SARS-CoV-2: An observational cohort study. Lancet Infect. Dis..

[183] Meng L., Hua F., Bian Z. (2020). Coronavirus Disease 2019 (COVID-19): Emerging and Future Challenges for Dental and Oral Medicine. J. Dent. Res..

[184] Bardellini E., Amadori F., Veneri F., Conti G., Majorana A. (2020). Coronavirus Disease-2019 and dental practice: A project on the use of ozonized water in the water circuit of the dental armchair. Stomatologija.

[185] Krishan K., Kanchan T. (2020). Aerosol and surface persistence: Novel SARS-CoV-2 versus other coronaviruses. J. Infect. Dev. Ctries..

[186] Martins-Filho P.R., de Gois-Santos V.T., Tavares C.S.S., de Melo E.G.M., do Nascimento-Junior E.M., Santos V.S. (2020). Recommendations for a safety dental care management during SARS-CoV-2 pandemic. Rev. Panam. De Salud Publica Pan Am. J. Public Health.

[187] Ghinai I., McPherson T.D., Hunter J.C., Kirking H.L., Christiansen D., Joshi K., Rubin R., Morales-Estrada S., Black S.R., Pacilli M. (2020). First known person-to-person transmission of severe acute respiratory syndrome coronavirus 2 (SARS-CoV-2) in the USA. Lancet.

[188] Zhao D., Yao F., Wang L., Zheng L., Gao Y., Ye J., Guo F., Zhao H., Gao R. (2020). A Comparative Study on the Clinical Features of Coronavirus 2019 (COVID-19) Pneumonia With Other Pneumonias. Clin. Infect. Dis..

[189] Day M. (2020). Covid-19: European drugs agency to review safety of ibuprofen. BMJ.

[190] Li J., Qiu Y., Zhang Y., Gong X., He Y., Yue P., Zheng X., Liu L., Liao H., Zhou K. (2021). Protective efficient comparisons among all kinds of respirators and masks for health-care workers against respiratory viruses: A PRISMA-compliant network meta-analysis. Medicine.

[191] Feng S., Shen C., Xia N., Song W., Fan M., Cowling B.J. (2020). Rational use of face masks in the COVID-19 pandemic. Lancet Respir. Med..

[192] Kariwa H., Fujii N., Takashima I. (2006). Inactivation of SARS coronavirus by means of povidone-iodine, physical conditions and chemical reagents. Dermatology.

[193] Kampf G. (2018). Efficacy of ethanol against viruses in hand disinfection. J. Hosp. Infect..

[194] Meeker H.G., Magalee R. (1986). The conservative management of the gag reflex in full denture patients. N. Y. State Dent. J..

[195] Eachempati P., Kumbargere Nagraj S., Kiran Kumar Krishanappa S., George R.P., Soe H.H.K., Karanth L. (2019). Management of gag reflex for patients undergoing dental treatment. Cochrane Database Syst. Rev..

[196] Meethil A.P., Saraswat S., Chaudhary P.P., Dabdoub S.M., Kumar P.S. (2021). Sources of SARS-CoV-2 and Other Microorganisms in Dental Aerosols. J. Dent. Res..

[197] Gurzawska-Comis K., Becker K., Brunello G., Gurzawska A., Schwarz F. (2020). Recommendations for Dental Care during COVID-19 Pandemic. J. Clin. Med..

[198] Hallam C., Denton A., Thirkell G. (2020). COVID-19: Considerations for the safe management and disposal of human excreta. Infect. Prev. Pract..

[199] Pascolo L., Zupin L., Melato M., Tricarico P.M., Crovella S. (2020). TMPRSS2 and ACE2 Coexpression in SARS-CoV-2 Salivary Glands Infection. J. Dent. Res..

[200] Dziedzic A., Wojtyczka R. (2021). The impact of coronavirus infectious disease 19 (COVID-19) on oral health. Oral Dis..

[201] Naqvi A.R., Schwartz J., Brandini D.A., Schaller S., Hussein H., Valverde A., Naqvi R.A., Shukla D. (2021). COVID-19 and oral diseases: Assessing manifestations of a new pathogen in oral infections. Int. Rev. Immunol..

[202] Fantozzi P.J., Pampena E., Di Vanna D., Pellegrino E., Corbi D., Mammucari S., Alessi F., Pampena R., Bertazzoni G., Minisola S. (2020). Xerostomia, gustatory and olfactory dysfunctions in patients with COVID-19. Am. J. Otolaryngol..

[203] Askin O., Altunkalem R.N., Altinisik D.D., Uzuncakmak T.K., Tursen U., Kutlubay Z. (2020). Cutaneous manifestations in hospitalized patients diagnosed as COVID-19. Dermatol. Ther..

[204] Riad A., Klugar M., Krsek M. (2020). COVID-19-Related Oral Manifestations: Early Disease Features?. Oral Dis..

[205] Xu J., Li Y., Gan F., Du Y., Yao Y. (2020). Salivary Glands: Potential Reservoirs for COVID-19 Asymptomatic Infection. J. Dent. Res..

